# Early Intervention with a Preformed Eruption Guidance Appliance After the Excision of Cemento-Ossifying Fibroma: A Case Report

**DOI:** 10.3390/children12030379

**Published:** 2025-03-18

**Authors:** Yeonjin Ju, Soyoung Park, Jonghyun Shin, Taesung Jeong, Eungyung Lee

**Affiliations:** 1 Department of Pediatric Dentistry, Dental Research Institute, Pusan National University Dental Hospital, Yangsan 50612, Republic of Korea; yeonjj94@pusan.ac.kr (Y.J.); syparkpedo@pusan.ac.kr (S.P.); jonghyuns@pusan.ac.kr (J.S.); tsjeong@pusan.ac.kr (T.J.); 2 Department of Pediatric Dentistry, School of Dentistry, Dental and Life Science Institute, Pusan National University, Yangsan 50612, Republic of Korea

**Keywords:** cemento-ossifying fibroma, eruption guidance appliance, myofunctional therapy, myofunctional training, case report

## Abstract

Background: Cemento-ossifying fibroma (COF) is a benign, non-aggressive fibro-osseous tumor in which normal bone is replaced by fibrous tissue containing collagen fibers, bone, or cementum-like material. Although COF is rare in children, its occurrence requires careful management due to its potential impact on tooth development and occlusion. Surgical excision is the widely used treatment; however, post-operative occlusal guidance is crucial, particularly in pediatric patients. Case report: This case report presents the early intervention using a preformed eruption guidance appliance (EGA) following the excision of COF in a 5-year-old boy. The patient exhibited premature loss of the primary canine and displacement of the devel-oping permanent tooth bud. After surgical excision, an EGA was applied to facilitate proper eruption of the successor teeth and optimize oral muscle function. Over a four-year follow-up period, the permanent teeth in the affected area erupted favorably, and intercuspal relationships improved during the transition from primary to mixed dentition. Conclusions: Given the limited research on the long-term effects of COF on developing dentition, long-term follow-up and additional studies are necessary to further evaluate its impact and the effectiveness of eruption guidance appliances in pediatric patients.

## 1. Introduction

Fibro-osseous lesions are benign osseous lesions in which the normal bone structure is replaced by fibroblasts containing collagen fibers and bony or cement tissues. Cemento-ossifying fibroma (COF) is a lesion included in the fibro-osseous category and is classified as a benign tumor by the World Health Organization (WHO) [1,2,3].

Radiographically, COF appears as a diffuse, ground-glass-like mixed density with clear borders. The lesions mostly exhibit slow expansion but can occasionally grow aggressively and require surgical resection for treatment. They can occur in children, adolescents, and the elderly, but they are most commonly observed in individuals between the ages of 20 and 50 [4,5,6]. COF exhibits a higher prevalence in females compared to males. While earlier studies have reported a female-to-male ratio of approximately 5:1, a more recent systematic review by Gautier et al. suggests a revised ratio of 1.6:1 [1,7,8].

Although previous case reports have documented occurrences of COF in pediatric and adolescent patients, the majority have focused on adolescent populations [4,5,6,8,9]. Consequently, there is a lack of research on whether the successor permanent teeth erupt normally after the surgical resection of the lesion.

Preformed elastomeric eruption guidance appliances (EGAs) are designed to regulate the strength of tongue and lip muscles, improve oral muscle function imbalance, and induce a normal occlusal relationship. These appliances help alleviate pressure from the lips, tongue, and masticatory and facial muscles, allowing teeth to erupt more freely [10,11].

However, it remains unknown whether EGAs have a direct impact on predicting individual growth and development or on the eruption and development of permanent teeth. They are primarily used for habit correction prior to the initiation of comprehensive orthodontic treatment.

This case report presents the surgical resection of COF that occurred in the left maxillary region of a 5-year-old boy. Additionally, it demonstrates a case in which an EGA was used after surgical resection to improve dental alignment and occlusal relationships.

## 2. Case Report

**Figure 1:**
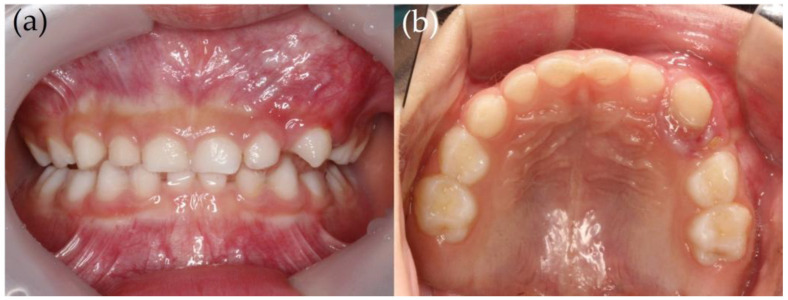
An intraoral photo of the patient at his initial visit. (**a**) Frontal view; (**b**) maxillary occlusal view. An indurated swelling lesion was observed on his maxillary left primary canine and primary first molar. He was asymptomatic except for the mobility of the maxillary left primary canine.

**Figure 2:**
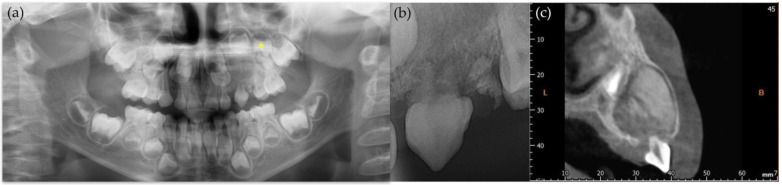
Radiographic photo of patient at his first visit. (**a**) Panoramic radiograph. Asterisk (*) refers to maxillary left first premolar; (**b**) periapical radiograph. Root of primary canine was shortened due to pathologic lesion; (**c**) coronal view of cone-beam computed tomography (CBCT). Well-defined mixed lesion on maxillary left primary canine area is shown with displacement of permanent teeth. L stands for lingual, and B stands for buccal.

A 5-year-old boy visited the Department of Pediatric Dentistry at the Pusan National University Dental Hospital with the swelling of the upper left gingiva and the resorption of an upper left deciduous tooth. According to the parents, the left upper deciduous canine showed gingival swelling right after the eruption; however, there was no pain. Upon initial examination, the clinical findings revealed gingival swelling and the resorption of the upper left deciduous canine, along with degree 3 mobility (Figure 1). Radiographic images, including periapical, panoramic, and cone-beam computed tomography, showed a mixed lesion with well-defined margins. Furthermore, because of the lesion, the displacement of the upper left deciduous molars in the distal direction was observed, and the tooth buds of the upper left canine and upper left premolars were also displaced (Figure 2).

Based on radiographic interpretation, a tentative diagnosis of ossifying fibroma was made. The upper left deciduous canine was extracted, and the lesion was surgically excised under general anesthesia. Excisional biopsy confirmed the diagnosis of COF.

At the 2-year follow-up examination, the natural movement of the permanent tooth buds in the upper left area was observed, but the anterior overjet and overbite were significant. The patient’s vertical facial pattern was normo-divergent, but due to the over-erupted maxillary anterior teeth and the retruded mandible, the patient exhibited a prominent mento-labial sulcus as a distinctive facial feature. The patient was evaluated as ‘positive’ according to the Frankl behavioral rating scale, which did not pose significant challenges to performing dental procedures in the clinic [12]. Moreover, after entering elementary school, he was rated as definitely positive, making the use of removable appliances entirely feasible. He was diagnosed with skeletal Class II malocclusion accompanied by the improper tooth positioning of the upper left first premolar after the orthodontic diagnostic examination. A preformed elastomeric eruption guidance appliance (EF line Class II standard, OrthoPlus Co., Igny, France) was selected for the patient, and myofunctional training was also planned simultaneously. If there was no improvement in the eruption of the maxillary left first premolar, comprehensive orthodontic treatment involving extraction was re-evaluated to resolve the maxillary protrusion.

**Figure 3:**
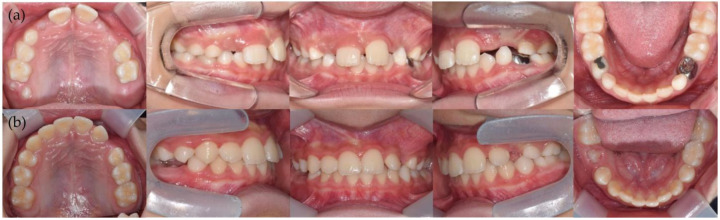
Changes in occlusion during the transition from primary to mixed dentition. Overbite and overjet were improved, and the dental arch was ideally shaped with an eruption guidance appliance (EGA). The photos were obtained when the patient was (**a**) 7 years old just before he started using an EGA and (**b**) 10 years old when he stopped using an EGA.

**Figure 4:**
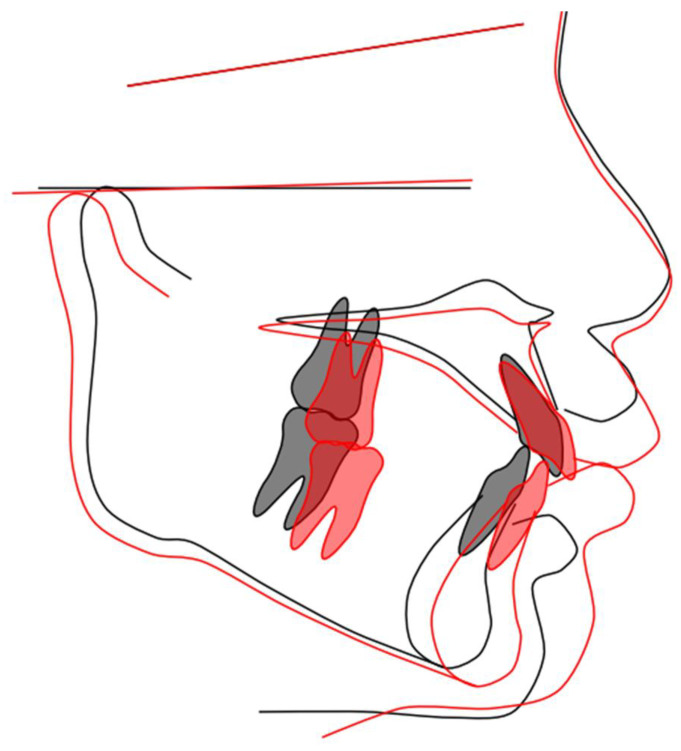
Lateral cephalograms. The treatment with EGAs was sustained for three. These radiographs were obtained when the patient was 7 years old (black line) and 10 years old (red line). The flattening of the occlusal plane, anterior mandibular growth, and the counterclockwise rotation of the mandible were observed. The lateral cephalogram tracing image was generated using WebCeph™ ver.2.0.0 (AssembleCircle Corp., Seongnam, Republic of Korea).

The patient showed good acceptance of the device and training, and after using the first EGA for approximately 14 months, the EGA was replaced with another (PreOrtho Type I-MH, PreOrtho, Tokyo, Japan). The appliance was sustained for three years. His upper and lower anterior teeth were positioned more favorably than before the treatment (Figure 3). Lateral cephalograms demonstrated the flattening of the occlusal plane following the treatment with the EGA, along with the counterclockwise rotation of the mandible and associated forward growth (Figure 4).

**Figure 5:**
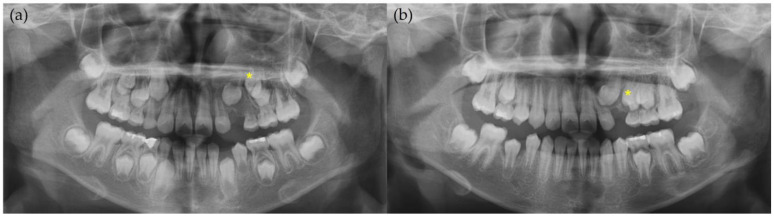
Panoramic radiographs during treatment. Asterisk (*) refers to maxillary left first premolar. Maxillary left canine developed well and erupted along its original eruption pathway. These radiographs were taken when patient was (**a**) 8 years old and (**b**) 10 years old.

At the 4-year follow-up examination, panoramic radiographs showed that the crown of the upper left canine was growing and erupting toward the root of the upper left lateral incisor and that the roots of the upper left first premolar were also developing. At the 4-year and 10-month follow-up examinations, panoramic radiographs showed that the crown of the upper left canine had erupted toward the root of the upper left lateral incisor and that the eruption direction of the upper left premolars had improved (Figure 5).

In the future, when the upper left primary molars are lost, it will be necessary to create space for the eruption of the upper left canine. A treatment plan for the eruption guidance of the upper left canine was established based on the orthodontic diagnosis. Therefore, follow-up appointments, including radiographic imaging for orthodontic diagnosis preparation, are scheduled regularly.

## 3. Discussion

COF primarily occurs in the mandible, specifically in the premolar and molar regions, and is more common in adults between the ages of 20 and 50, with a higher incidence in females [8,13]. However, in this case, the lesion occurred in the maxillary canine region, which is rare, especially considering the patient’s age of only 5 years at the time of their first visit. According to some studies, COF occurring in the maxilla tends to be more aggressive and present with more pronounced clinical symptoms and signs than lesions in the mandible [14]. This could be attributed to the pathological differences in the anatomical structures of the maxilla and mandible. Due to its thick cortical bone, the mandible provides a less favorable environment for lesion growth and expansion. In contrast, the maxilla has thinner cortical bone, allowing the lesion to expand more easily from the cancellous bone.

There are no official recommendations established for the management of COF. In general, conservative surgical approaches such as enucleation, curettage, and lesion excision are suggested for small lesions, whereas radical surgery with reconstruction may be required for giant, recurrent, or aggressive lesions [8]. The recurrence rate of ossifying fibromas is reported to be 12–28% [15]. However, the recurrence rate of COF specifically has not been widely documented. A systematic review by Gautier et al. estimated the recurrence rate of COF to be approximately 9% [8]. The timing of recurrence of the lesion varies from 6 months to 7 years, highlighting the need for long-term follow-up exceeding 10 years [16,17].

Juvenile COF (JCOF) shares similar pathological characteristics with COF but is clinically more aggressive and primarily occurs in individuals under the age of 15 years. JCOF is characterized by a wider range of involvement and induces a significant expansion of the cortical bone, necessitating extensive resection, including the affected teeth and permanent dentition [16]. In this case, JCOF was excluded because of the relatively small size of the lesion and its non-aggressive growth pattern [18]. As COF mostly occurs in adults, there is a lack of studies on the eruption and alignment of permanent teeth in areas affected by COF. JCOF, which frequently occurs in children, requires extensive resection, including that of the permanent tooth buds. Therefore, research on whether permanent tooth eruption and normal growth occur postoperatively is limited. Tortorici et al. reported that JCOF involving the mandibular second premolar region resulted in eruption disturbances and positional changes. After the surgical enucleation of the lesion, the spontaneous eruption of the teeth and improvement in the direction of eruption occurred [19]. This case report demonstrates the growth and eruption of adjacent permanent teeth after the resection of a relatively small COF. Further research is required on the eruption and alignment of permanent teeth in areas affected by COF.

Since the patient was only 5 years old at the time of surgery, long-term follow-up was essential for occlusion management due to the early loss of deciduous canine and to ensure the normal development and eruption of permanent successors. Orthodontic evaluation was performed after 2 years of the COF excision. He was diagnosed with dentoskeletal Class II malocclusion with increased overjet and overbite. The tooth bud of the maxillary left first premolar was positioned unfavorably. Two EGAs were used to improve the maxillary relationship while waiting for the normal eruption time of the maxillary premolars. The patient showed high compliance with the appliance, and an improvement in the anterior overjet and overbite was established. Furthermore, the eruption path of the maxillary left premolar was changed for the better.

In the treatment of Class II malocclusion in mixed dentition children, appliances such as an activator or bionator and Twin Block are traditionally used [20,21]. These appliances have been in use for a long time and are known to provide reliable outcomes; however, they require impression-taking and a laboratory fabrication process. In contrast, EGAs demonstrate a bite correction effect comparable to that of traditional appliances but offer superiority in terms of convenience. Since these appliances can be selected chairside according to the arch size, they eliminate the need for impression-taking, laboratory procedures, and additional patient visits. Another advantage of EGAs is that they can enable longer check-up intervals and shorter chair times at each visit [22].

EGAs have been used in Europe since the 1930s [10]. These appliances aim to improve the imbalance in oral muscle function by controlling the forces exerted by muscles on the basal bone and dental arches [23]. In cases of skeletal Class II malocclusion, where the maxilla is narrow, restricting the growth of the mandible and causing it to be positioned posteriorly, EGAs help relieve the forces exerted by the muscles on the maxilla, allowing for the expansion of the maxillary arch and the forward positioning of the mandible [24]. When such treatment is performed during the mixed dentition stage, it can be useful in reducing the need for tooth extraction or orthognathic surgery during permanent dentition [10]. Additionally, the use of EGAs in pediatric and adolescent patients with skeletal Class II malocclusion can lead to the forward movement of the mandible, expansion of the airway, and facilitation of better nasal breathing [25].

Recent studies have reported that EGAs are effective in Class II treatment and have good short- and long-term stability [26,27,28]. The stability of post-treatment outcomes is closely related to the severity of the pre-treatment condition [29]. An improper anteroposterior dimension may allow incisors to over-erupt, potentially leading to the recurrence of a deep bite. In particular, if an EGA is used in the early mixed dentition period, growth modification can be induced to achieve a good alignment of the maxillary incisors, and it leads favorable overjet and overbite [22,26,30]. This highlights the importance of early treatment in Class II patients to advance the retruded mandible, preventing the extrusion of maxillary incisors and establishing a normal overjet and overbite. It can also improve the molar relationship and maintain a stable occlusion with only phase 1 treatment and is as effective as traditional functional devices [27,31].

However, EGAs have several limitations. EGAs facilitate the guidance of tooth eruption into a favorable position; they do not exert direct forces to reposition or realign teeth. Therefore, the regular monitoring of the eruption pathway is essential throughout treatment. In cases where ectopic eruption is pronounced, more comprehensive orthodontic intervention may be necessary to achieve optimal dental alignment.

Patient compliance is a key factor in determining the success of removable orthodontic appliances [32]. In this case, the patient demonstrated excellent cooperation, posing no challenges for the pediatric dentist in terms of compliance. Although children who received EGA treatment in early mixed dentition may not require a second treatment phase, in this case, fixed orthodontic treatment is planned to guide the eruption of the maxillary left canine and first premolars.

## 4. Conclusions

COF is a rare disease that requires surgical excision in children. It can be diagnosed using clinical, radiological, and pathological examinations. When COF occurs in children, long-term follow-ups are necessary for permanent tooth eruption and occlusal management after surgical excision.

This case report highlights the successful use of a preformed EGA after COF excision in a 5-year-old child, demonstrating its effectiveness in facilitating proper tooth eruption and occlusal development over a four-year period. The EGA contributed to favorable tooth alignment, improved intercuspal relationships, and enhanced oral muscle function. However, regular monitoring may be necessary. This case underscores the importance of early intervention and long-term follow-up in pediatric COF cases. Further research is needed to evaluate occlusal management after COF excision.

## Data Availability

The original contributions presented in this study are included in the article. Further inquiries can be directed to the corresponding author.

## Abbreviations

The following abbreviations are used in this manuscript:

COF	Cemento-ossifying fibroma
WHO	World Health Organization
EGA	Eruption guidance appliance
JCOF	Juvenile cemento-ossifying fibroma
